# Community-Based Lifestyle Intervention for Reducing Blood Pressure and Glucose among Middle-Aged and Older Adults in China: A Pilot Study

**DOI:** 10.3390/ijerph111111645

**Published:** 2014-11-13

**Authors:** Aihua Lin, Guanrong Zhang, Zhiting Liu, Jing Gu, Weiqing Chen, Futian Luo

**Affiliations:** 1 Department of Medical Statistics and Epidemiology, School of Public Health, Sun Yat-sen University, No. 74 Zhongshan 2nd Road, Guangzhou 510080, China; E-Mails: linaihua@mail.sysu.edu.cn (A.L.); liuzhiting6@163.com (Z.L.); gujing0828@hotmail.com (J.G.); chenwq@mail.sysu.edu.cn (W.C.); 2 Health Management Center, Guangdong General Hospital, Guangdong Academy of Medical Sciences, No. 123 Huifu Xi Road, Guangzhou 510180, China; E-Mail: gavincheung@yeah.net

**Keywords:** hypertension, diabetes mellitus, lifestyle intervention, community, middle-aged and older adults, China

## Abstract

Although evidence suggests that lifestyle interventions can reduce blood pressure (BP) and glucose levels, there is little information about the feasibility of such interventions when implemented in community settings. This study evaluated the effectiveness of a community-based lifestyle intervention on BP and glucose in the middle-aged and older Chinese population. By using a cluster randomisation approach, 474 participants from two communities were assigned to the intervention group which received intensive health education and behavioural intervention, or the control group which received conventional education. Linear mixed models were used to compare between-group differences on change in BP and fasting glucose after 6, 12 and 24 months. At the 12-month follow-up, the intervention group experienced significantly reductions in systolic BP (−4.9 *vs.* 2.4 mmHg; mean difference [MD] −7.3 mmHg; *p* < 0.001), diastolic BP (−1.9 *vs.* 1.9 mmHg; MD −3.8 mmHg; *p* < 0.001) and fasting glucose (−0.59 *vs.* 0.08 mmol/L; MD −0.67 mmol/L; *p* < 0.001). These differences were sustained at the 24-month follow-up. With only two communities, it was not possible to adjust for potential clustering by site. This approach of lifestyle interventions conducted through primary care services may be a potential solution for combating hypertension and diabetes in a resource-limited country context in China.

## 1. Introduction

Cardiovascular disease (CVD) and diabetes mellitus (DM) have become the leading global causes of death in adults, collectively accounting for 1 in 4 deaths [1]. High blood pressure (BP) and glucose are the greatest risk factors for death and disease associated with CVD [2,3]. According to Chinese national data, approximately 256.2 million adults had hypertension (HT) and 92.4 million adults suffered from diabetes in 2007 [4,5], substantially increasing their risk of developing CVD. Because the prevalences of HT and DM are expected to continue to increase in China in the next two decade [6], interventions targeting BP and glucose control are urgent public health priorities.

Substantial evidence from randomized controlled trials has demonstrated that lifestyle intervention consisting of weight loss, dietary modification and increased physical activity can effectively lower BP and glucose [7,8] and improve CVD-related outcomes [9,10]. However, these trials involved enrollment criteria and intensive lifestyle interventions that are challenging to replicate in community primary care settings [11,12]. Because such healthcare settings have a limited capacity to offer intensive lifestyle interventions, which need rather much work from the providers and is not a very low-cost and easy primary care service. In this context, there is an urgent need to translate this evidence into real-world community settings. Unfortunately, findings from high-income countries are inconclusive on the effectiveness of community-based lifestyle interventions for lowering BP and glucose [13]. Reports of health promotion programmes targeting BP and/or glucose reduction in countries with lower resources, such as China, remain limited. Additionally, because high BP and glucose interact multiplicatively in the development of CVD, an intervention proven to simultaneously improve BP and glucose control in a community setting could be widely adopted, with the potential to significantly reduce the public health burden of CVD.

Although current national guideline for the prevention and control of HT and DM emphasise health education and the availability of lifestyle interventions through primary care services, the provision of such services in most community clinics is inadequate. Moreover, studies have indentified gaps between the guideline and community physicians’ knowledge and practice regarding HT [14,15]. Therefore, our objective was to determine whether a community-based lifestyle intervention delivered by field health workers for middle-aged and older adults in an urban limited-resource community can be effective in reducing BP and glucose, and whether any beneficial effects can be sustained in the long term.

## 2. Methods

### 2.1. Study Setting

The community-based lifestyle intervention study was conducted from January 2010 to March 2012 in two communities of Yuexiu district, located in the center of Guangzhou city, China. Yuexiu district covers 22 neighborhoods (the primary administrative area of a city in China, which consists of several communities) and has a population of about 1.16 million, 29.7% of which are aged 50 and above. Within the district, there are 22 community health clinics (one for each neighborhood) providing primary care services for all local inhabitants. The prevalences of HT and DM are estimated to be 21.9% and 6.6% among the adult population, respectively.

This community-based study involved a collaboration between the local residential committees, community health clinics and the study team from the School of Public Health, Sun Yat-sen University. Most study activities, including screening, enrolment, and group intervention, were carried out by field health workers at the community health clinics. Key members of the research team provided staff training, intervention support and materials and built relationships with the community.

### 2.2. Study Design

The study followed a cluster randomized controlled trial design, in which interventions were allocated at the community rather than the individual level to minimise contamination among participants within the same community. Using data from a 2009 statistical bulletin of national economic and social development in Yuexiu district, a convenience sampling of two neighborhoods with similar demographic and socioeconomic characteristics were selected. Invitation letters were sent to two community health clinics located in the selected neighborhoods, requesting their participation in the study. These two clinics (Zhuguang and Liurong), consisting of 18 and 10 communities, were willing to participate. Finally, one community within each neighborhood was randomly selected and assigned to deliver a community-based lifestyle promotion programme or conventional health education. This community is the primary service area of the community clinic and served as the study’s base location. The randomisation was done via a computerized procedure by an independent statistician. The distance between the intervention and control sites was 5 km. The study participants were allocated to the intervention or control group depending on which community clinic they were enrolled at the time of the baseline survey.

### 2.3. Ethics Statement

The study protocol was approved by the Institutional Review Board of the School of Public Health, Sun Yat-sen University. Written informed consent was obtained from all the study participants. Data was preserved in Department of Medical Statistics and Epidemiology, Sun Yat-sen University.

### 2.4. Participants and Recruitment

The enrolment and baseline survey took place from January to March 2010. Participants were recruited via a community-wide campaign and advertisements, including informed consent letters, posters and telephone contact details, distributed by the residential committee. The field health workers made public service announcements during their clinics, inviting eligible patients to participate. The participants met the following inclusion criteria: (1) aged 50 to 79 years; (2) registered as a resident of Guangzhou and had lived in the study community for at least 6 months; (3) able to provide written informed consent to participate in the study. The exclusion criteria were: (1) prior cardiovascular events, chronic kidney disease, diabetic foot, secondary HT, cancer, mental illness or poor vision or hearing; (2) currently participating in another clinical trial or had done so within the previous 6 months; (3) intended to leave the study community in the coming two years. According to the baseline screening, the participants in each group were classified as non-hypertensive or hypertensive following the definition of HT [16], so that specific intervention components could be targeted and the effects among subgroups compared.

### 2.5. Intervention Group

A 12-month lifestyle promotion programme was administered through the existing community-based system for the management of HT and DM. The intervention components were developed by a panel consisting of two health education experts, a cardiologist, two field health workers and a hypertensive patient. The framework was based on the health belief model (HBM), which hypothesises that a particular form of behaviour depends on the individual’s personal beliefs about the perceived threat posed by a health problem, together with the effectiveness of the proposed behavioural change in reducing the threat at an acceptable cost [17]. The contents were derived from the PREMIER trial [18], in which a comprehensive lifestyle intervention programme including dietary change and increasing physical activity that successfully improved lifestyle behaviours and lowered BP. An overview of the intervention framework is outlined in Table 1.

**Table 1:** Overview of intervention activities and healthy lifestyle information.

Construct	Activity	Content
Perceived susceptibility	Basic education on HT and DM	Definitions and diagnosis of HT and DM; risk factors and high-risk subjects for CVDs; living a healthy lifestyle and the association with disease; main modifiable behavioural risk factors (BRFs)
	Cardiovascular risk stratification and information provided to participants	Classification of BP and fasting plasma glucose (FPG) levels; high-risk-group screening for normal BP and FPG participants; cardiovascular risk and subclinical organ damage assessment for HT and DM participants; participants informed of the evaluation results
Perceived severity	Extended education on HT and DM	Prevalence and disease burden of HT, DM and their cardiovascular complications; lifestyle and behavioural risk factors (including obesity, smoking, excess alcohol intake, unhealthy diets, physical inactivity, medication non-adherence) and the short- and long-term consequences
Perceived benefits	Guidance of lifestyle modification	Preventability of HT, DM and CVDs; health benefits of reducing BRFs; appropriate methods for changing and maintaining specific BRFs (including weight loss, smoking cessation, reduction in daily alcohol consumption, sodium and fat intake, increase in daily intake of vegetables and fruits, importance of engaging in physical exercise and tips for treatment adherence)
	Behavioural skills training	Self-monitoring of weight, BP and FPG; diary recording of main BRF changes; developing a schedule and action plan
	Regular BRFs and health assessment	Schedule clinical contacts and health review; feedback on the assessment
Perceived barriers	Training and Counselling	Identifying obstacles; problem-solving and decision-making skills; information and social support for coping with negative events and seeking assistance
Cues to action	Material delivery and phone contact	Self-education materials for HT and DM management (pamphlet, textbook, films and educational prescription); phone contacts for information support and follow-up reminder
Self-efficacy	Goal-setting and behavioural training	Provide achievable short-term goals for behavioural changes and BP, FPG control; regular follow-ups; check the status of goal attainments; behavioural training for goal-setting and self-reinforcement

The lifestyle intervention consisted of a common component of sixteen 1-h group sessions for all participants, and a specific component of six 30-min individual sessions for patients with HT and/or DM. Both components were tailored for individual participants by five field health workers. The group sessions were held at an easily accessible community site (the Starlight Home for the Elderly) and individual sessions were conducted at the clinic. The overall sessions were divided into two stages, each with specific goals. An instruction manual was produced to guide the implementation of the intervention.

The first stage ran from April to September 2010. It was made up of eight information sessions and two counselling sessions, delivered in a group format. The first two study sessions aimed to build rapport and to identify participants’ knowledge gaps with regard to HT and DM prevention, beliefs about prevention and barriers to making lifestyle changes. The other six study sessions were designed to address the identified knowledge gaps (HT and DM are strongly associated with unhealthy lifestyles and can be prevented and controlled by lifestyle changes), beliefs about disease prevention and lifestyle changes (confidence in the ability to control HT and DM or facilitate lifestyle changes) and anticipated challenges and solutions to making changes. Information provision targeted the importance of increasing physical exercise, decreasing the use of salt and cooking oil and increasing vegetable and fruit consumptions, maintaining normal body weight, quitting smoking, reducing excessive alcohol intake, and adhering to pharmacological treatment. At the end of each session, participants were encouraged to form action plans and set achievable goals for making lifestyle changes. Participants attended the first session a week after completing the baseline survey and subsequent sessions every 2 weeks. The counselling sessions were designed to enhance their self-efficacy in initiating lifestyle changes. Participants were asked to assess their goal attainment and discuss difficulties and countermeasures in making lifestyle changes. The discussions aimed to adapt participants’ expectations about lifestyle changes by correcting unrealistic thoughts and promoting positive attitudes. Afterwards, participants reviewed and revised their previous action plans with the assistance of the field health workers. The counselling sessions were delivered on a monthly basis after the completion of the information sessions.

The second stage (from October 2010 to March 2011) consisted of six group-based interactive training sessions. First, these sessions aimed to provide skills and guidance in accomplishing lifestyle changes. The training activities included demonstrations of healthy dietary patterns and cooking methods, participating in regular physical exercise and resistance and relapse prevention skills for smoking and alcohol use during the sessions; Second, participants were offered inducements to encourage them to adopt a healthy lifestyle (e.g., a set of 6-g salt spoons and a 50-g oiler, sample recipes for culturally acceptable and economically feasible foods and a customised physical exercise regime); Third, reinforcements and support were provided to strengthen participants’ self-efficacy in maintaining a healthy lifestyle. Participants were encouraged to establish realistic goals and to keep a diary of their dietary intake and physical exercise. At each session, participants rated their goal attainment, discussed barriers and solutions to achieving their goals, and set a new goal and action plan with the help of field health workers; Fourth, participants discussed both their positive and negative experiences related to the lifestyle changes and to their lives in general; Lastly, further efforts were made to emphasise that lifestyle changes require long-term adherence (e.g., reminder calls or messages were used to motivate participants in adopting the target lifestyle). The training sessions in this stage were held once a month. 

During the second stage, patients with HT and/or DM received another six individual education sessions on pharmacological treatment. The sessions were delivered at monthly intervals and included (1) the importance of achieving recommended targets (BP < 140/90 mmHg, FPG < 7.0 mmol/L) and adhering to medications; (2) information about the adverse effects of prescribed medicines and how to cope with them; (3) rectifying medication-related misconceptions and forming positive and realistic outcome expectations regarding the usage of medications; (4) reviewing patients’ anti-hypertensive and/or diabetic medications and providing reasonable regimens in accordance with recent guidelines; (5) discussing the challenges to medication adherence and feasible solutions. However, the study did not provide medications or fee-paying services.

### 2.6. Control Group

Control group participants received only the conventional health education for common chronic diseases. Twelve 40 health lectures were provided by a local general physician on a monthly basis. The lectures included basic knowledge about HT, DM, dyslipidaemia, fatty liver disease, cardiovascular disease, cancer, chronic respiratory diseases, digestive diseases and osteoporosis. Two additional activities were held on the HT and DM Health Days (e.g., volunteer medical consultations and disease prevention posters). Apart from such activities, control group participants with HT or DM received the routine conventional diagnosis and treatment services offered by the community health clinic, but no other lifestyle promotion services.

### 2.7. Training of Field Health Workers

A team of five Cantonese-speaking field health workers (three physicians and two nurses) delivered the intervention. All of the health workers had a post-high school degree and had worked in a cardiac or diabetic clinic for more than 5 years. An 8-h training workshop was organised to ensure they understood the intervention protocol, including the lifestyle and pharmacological aspects and the theological framework, and to deliver training in interpersonal communication, motivational counselling and behavioural intervention techniques. The training focused on standardised operational procedures for community-based prevention and management of HT and DM, based on the Chinese guidelines for HT and DM [16,19]. This session used a case-based approach involving role-playing and mock practice and was conducted by a health education expert and a cardiologist. At the end of the workshop, a certificate of training was provided to the trainees.

### 2.8. Follow-Up Procedures

All participants were followed-up at 6, 12 and 24 months after completion of the baseline survey. To maximise retention, reminders were sent out two days before the scheduled follow-up. At each visit, participants completed a routine physical examination, which was consistent with the protocol used at baseline. Information on changes in lifestyle and use of anti-hypertensive and/or diabetic medications was also collected. All of the assessments were performed by well-trained outcome assessors, who were medical students and unaware of the intervention allocation. All measures took place during the first half of the day and balanced between groups to minimise diurnal variations in BP. Participants were reimbursed 20 RMB for the baseline survey and for each of the three follow-up visits.

### 2.9. Outcome Measures and Classification

Physiological measures included BP, FPG, body weight and height. First, BP was measured with an automatic Omron sphygmomanometer in a sitting position after 5 min of rest, and the mean of two measures taken 2 min apart was used. HT was defined as systolic BP ≥ 140 mmHg and/or diastolic BP ≥ 90 mmHg, and/or currently taking anti-hypertensive medications; Second, blood samples were collected after a 10-h overnight fast, and plasma glucose was measured using the hexokinase enzymatic method. Participants with an FPG level ≥ 7.0 mmol/L and/or self-reported current use of anti-diabetic medications were diagnosed as DM cases; Third, two sets of anthropometric measurements were taken with participants wearing light clothing and no shoes. Body weight and height were measured using a scale and a wall-mounted stadiometer, to the nearest 0.5 kg and 0.5 cm, respectively, and were used to calculate BMI (kg/m^2^).

Self-reported behavioural data, including smoking, alcohol use, diet, physical exercise and medication adherence, were obtained using standard questionnaires. First, current smoking was defined as at least one cigarette a day for 6 months or more; Second, alcohol use was defined as the consumption of at least one alcoholic drink per week for 1 year or more; Third, dietary intakes were assessed by a semi-quantitative food frequency questionnaire of 49 food items, which was specifically developed for the Chinese population [20] and has been validated against a series of food weighted records and 24-h dietary recall [21]. The participants were asked how frequently and what quantity of certain foods or food groups they consumed; Fourth, physical exercise was estimated based on recall of a typical week, following the WHO’s STEP approach [22], and categorised into three levels: active (≥30 min per day at least 3 days a week of moderate or vigorous activity), somewhat active (exercise but not at active level) and inactive (have no exercise); Finally, medication adherence was assessed using the 4-item self-reported Morisky Medication Adherence Scale [23], on which patients were classified as adherent or non-adherent.

Outcome analyses focused on changes in systolic BP (primary outcomes), and diastolic BP, FPG, BMI and lifestyle variables (secondary outcomes). The protocol specified a primary assessment time-point of 12 months to measure the immediate effectiveness of the intervention. The final visit occurred at 24 months to assess the long-term sustainability of the intervention. Few lifestyle intervention studies have included a 2-year follow-up period.

### 2.10. Sample Size

We determined the sample size using information about expected reductions in systolic BP and the correlation structure of repeated measurements within participants over time. The PREMIER revealed a difference of 3.7 to 4.3 mmHg in systolic BP at 6 months, and a cross-sectional SD of 9.6 mmHg [18]. We assumed a modest correlation of *r* = 0.2. Therefore, the SD for the follow-up measure after adjusting for the baseline value was calculated as 9.6 × the square root of (1 − 0.2^2^) = 9.406. These values were entered into the Stata program to calculate the sample size for a two-group trial, with a type-I error rate of 0.05. Accordingly, 177 subjects were required in each group to achieve 85% power to detect a difference of 3 mmHg in systolic BP in the entire sample at 12-month follow-up. Assuming a 30% dropout rate, the total number of participants was calculated as 462 (231 per group).

### 2.11. Statistical Analyses

For baseline comparison between the intervention and control groups, *t*-tests were used for continuous variables and Chi-square tests were used for categorical variables. We calculated 6-, 12- and 24-month change in the outcome measures from the baseline to follow-ups. To estimate mean changes in the primary and secondary outcomes, linear mixed models for repeated measures were used, taking into account the correlations between individuals’ repeated measures over time. Fixed terms for the baseline measurements, allocation group, visit and group × visit interaction were included in the models. As the groups were found to be imbalanced on several variables at baseline, we calculated marginal mean differences with 95% confidence intervals (CI) for the intervention effects, adjusted for potential confounders. Furthermore, we conducted subgroup analyses for the efficacy parameters after stratification by participants’ baseline BP status. Consistent with the intention-to-treat principle, the analyses included all available data for participants who received the intervention activities and had completed the baseline and at least one follow-up assessment. Our analyses techniques made an assumption of ignorable dropout and missing data were not imputed. Because this was a pilot study involving only two allocation communities, it was not possible to adjust for potential clustering by site.

We also conducted sensitivity analyses on the completion population, including all participants for whom data were available at all study assessments. The mixed-effect logistic models were used to estimate odds ratios in health-related behavioural variables between the intervention and control groups. All statistical analyses were carried out in SAS, version 9.1 (SAS Institute, Cary, NC, USA). Two-sided *p* values are reported with a statistical significance level of <0.05.

## 3. Results

**Figure 1:**
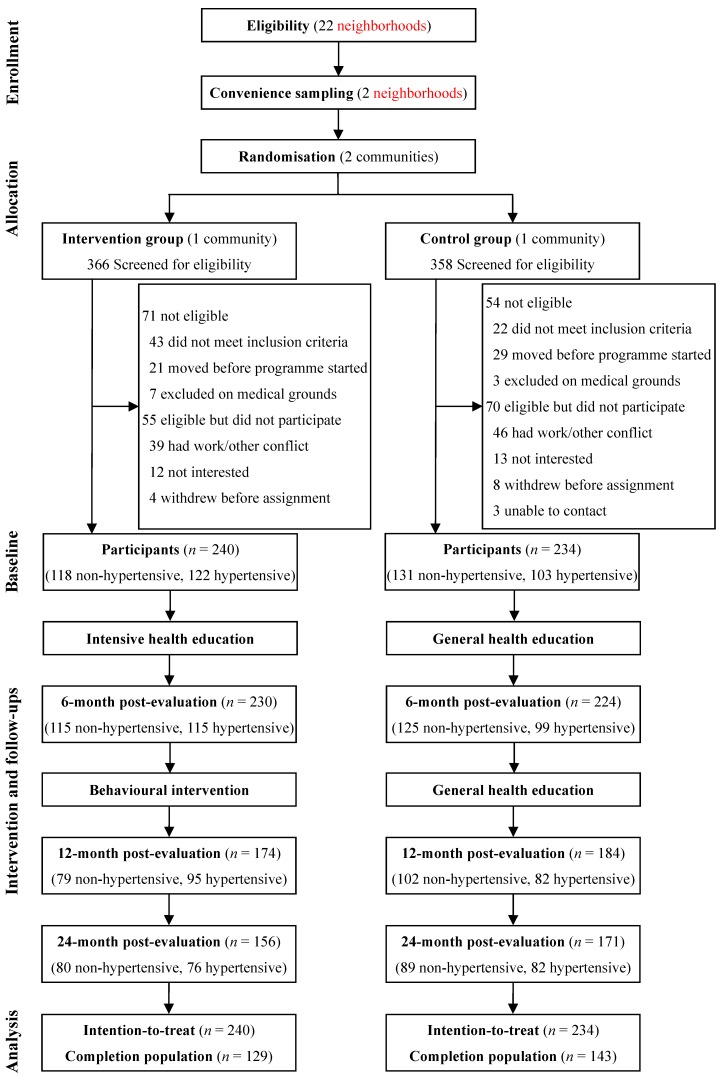
Study design and participant flow.

Of the 724 residents who registered with the residential committee and were screened for eligibility, 125 were found to be ineligible and 125 were excluded according to the exclusion criterion. The participation rate was 79%, which was higher in females in the control group (80% *vs.* 70%, *p* = 0.061) but not in the intervention group (80% *vs.* 83%, *p* = 0.562). The remaining 474 participants completed the baseline assessment and were assigned to the intervention group (*n* = 240) or the control group (*n* = 234) according to the community allocation, and were included in the analyses (Figure 1).

### 3.1. Baseline Characteristics and Comparison between Groups

The mean (SD) age of participants was 58.2 (7.0) years, 65.8% were female, 56.5% had a high school education or above and 63.1% had low incomes (defined as average household income less than 2000 RMB per month). Mean (SD) systolic and diastolic BP were 133.3 (14.4) and 80.5 (8.7) mmHg, and the mean (SD) FPG was 6.2 (1.4) mmol/L. The baseline prevalences of HT and DM were 47.5% and 15.4%, respectively.

The characteristics of the two groups were similar in most demographic and baseline measurements (Table 2 and Table 3), although participants in the control group were more likely to be female (70.5% *vs.* 61.2%), younger (57.5 *vs.* 58.8 years), have higher systolic BP (134.6 *vs.* 132.0 mmHg) and have a higher intake of vegetables (485.8 *vs.* 386.4 g/d).

**Table 2:** Demographic and baseline characteristics of the participants.

Variable	Intervention (*n* = 240) *n*(%) or mean ± SD	Control (*n* = 234) *n*(%) or mean ± SD	*p*
Female	147 (61.2)	165 (70.5)	0.034
Age, year	58.8 ± 7.9	57.5 ± 6.0	0.044
Education level			0.253
Secondary school or lower	110 (45.8)	96 (41.0)	
High school	111 (46.3)	115 (49.2)	
College or above	19 (7.9)	23 (9.8)	
Married	201 (83.8)	199 (85.0)	0.698
Low income	158 (65.8)	141 (60.3)	0.208
Family history of HT	117 (48.8)	128 (54.7)	0.195
Family history of DM	45 (18.8)	52 (22.2)	0.349
Current smoker	44 (18.3)	30 (12.8)	0.098
Alcohol use	32 (13.3)	26 (11.1)	0.460
Dietary intake			
Salt, g/d	8.3 ± 2.7	8.4 ± 2.1	0.831
Cooking oil, g/d	25.8 ± 6.8	26.0 ± 6.5	0.791
Vegetables, g/d	386.4 ± 170.7	485.8 ± 184.3
Fruits, g/d	108.3 ± 65.9	114.0 ± 58.9	0.324
BMI, kg/m^2^	23.8 ± 3.2	23.8 ± 2.7	0.997
SBP, mmHg	132.0 ± 14.9	134.6 ± 13.8	0.046
DBP, mmHg	80.9 ± 9.0	80.0 ± 8.4	0.250
FPG, mmol/L	6.21 ± 1.29	6.27 ± 1.50	0.609
Hypertensive	122 (50.8)	103 (44.0)	0.137
Diabetic	36 (15.0)	37 (15.8)	0.807

### 3.2. Follow-Up of Participants

Overall, the follow-up attendance rate was 95.8% at 6 months, 75.5% at 12 months and 69.0% at 24 months, with no substantive difference between group allocations. However, among hypertensive patients, the 24-month retention rate was significantly higher in the control group than in the intervention group (79.6% *vs.* 62.3%, *p* = 0.005). Overall, 272 (57.4%) participants completed all three follow-up visits: 129 in the intervention group, and 143 in the control group.

There were no significant differences in the baseline characteristics of missing and retained samples at follow-ups, except that younger participants were more likely to drop out at 12 and 24 months (*p* < 0.05). Participants who had a lower BMI (23.3 *vs.* 24.0 kg/m^2^, *p* = 0.038) at baseline were also more likely to drop out at 12-month follow-up.

### 3.3. Intervention Attendance

The intervention participants attended, on average, 13 of the 16 group sessions over the 12-month period; 92% of participants attended the first-stage intervention and 69% attended the second-stage training. Patients with HT and/or DM in the intervention group attended a median of four additional individual sessions during the second-stage intervention. The attendance rate at individual sessions was 73%.

### 3.4. Changes in Primary and Secondary Outcomes

The mean changes in BP, FPG and BMI profiles over the course of the 24-month study are listed in Table 4. BP declined progressively during the 12-month intervention period in the intervention group, but this improvement was not sustained at the final visit. In contrast, the control group presented significant increases in both systolic and diastolic BP at follow-up. The mean differences (adjusted for sex, age, education and baseline BP) in systolic BP between the intervention and control group participants at 6, 12 and 24 months were −5.4, −7.3 and −3.9 mmHg, respectively (all *p* < 0.001). The corresponding differences in diastolic BP were −3.4, −3.8 and −2.3 mmHg, respectively (all *p* < 0.001).The between-group differences in both systolic (3.4 mmHg, 95%CI 1.0 to 5.8, *p* = 0.005) and diastolic (1.5 mmHg, 95%CI −0.1 to 3.1, *p* = 0.069) BP were significantly less at 24 than at 12 months.

Similar patterns were observed for changes in FPG and BMI over time (Table 4). Relative to the control group, the FPG in the intervention group decreased by −0.38, −0.67 and −0.23 mmol/L at 6, 12 and 24 months, respectively (all *p* < 0.001). The intervention group participants also showed significant decreases in BMI compared with the controls over time (6 months: −0.28 kg/m^2^; 12 months: −0.50 kg/m^2^; 24 months: −0.38 kg/m^2^; all *p* < 0.001). Further analyses showed that the differences between the two groups in terms of reduction in FPG and BMI were greater at 12 months that at 6 months (FPG: 0.29 mmol/L, 95%CI 0.09 to 0.50, *p* = 0.005; BMI: 0.22 kg/m^2^, 95%CI 0.08 to 0.37, *p* = 0.002). Sensitivity analyses conducted on the completion population yielded consistent results for the primary and selected secondary outcomes (Table A1).

### 3.5. Subgroup Analyses

Table 4 displays the specified subgroup analyses stratified by baseline BP status. In all participants, both non-hypertensive and hypertensive, the changes in systolic and diastolic BP were consistent with the main findings. In contrast with the control group, the interventions substantially reduced systolic and diastolic BP in both subgroups. Similar results were observed for absolute changes in FPG and BMI.

**Table 3:** Number (%) of participants in the two assigned groups by activity and use of HT medication category at baseline and follow-up.

Variable	Intervention				Control			
	Baseline	6 months	12 months	24 months	Baseline	6 months	12 months	24 months
Leisure physical activity	(*n* = 240)	(*n* = 230)	(*n* = 174)	(*n* = 156)	(*n* = 234)	(*n* = 224)	(*n* = 184)	(*n* = 171)
Inactive	152 (63.3)	64 (27.8)	36 (20.7)	45 (28.8)	148 (63.2)	126 (56.3)	102 (55.4)	88 (51.5)
Somewhat active	39 (16.3)	47 (20.4)	30 (17.2)	30 (19.2)	21 (9.0)	41 (18.3)	29 (15.8)	31 (18.1)
Active	49 (20.4)	119 (51.7)	108 (62.1)	81 (51.9)	65 (27.8)	57 (25.4)	53 (28.8)	52 (30.4)
Medications for HT	(*n* = 122)	(*n* = 115)	(*n* = 95)	(*n* = 76)	(*n* = 103)	(*n* = 99)	(*n* = 82)	(*n* = 82)
No medication	58 (47.5)	21 (18.3)	20 (21.1)	13 (17.1)	55 (53.4)	34 (34.3)	28 (34.1)	25 (30.5)
Non-adherence	29 (23.8)	30 (26.1)	13 (13.7)	27 (35.5)	23 (22.3)	39 (39.4)	24 (29.3)	30 (36.6)
Adherence	35 (28.7)	64 (55.7)	62 (65.3)	36 (47.4)	25 (24.3)	26 (26.3)	30 (36.6)	27 (32.9)

**Table 4:** Mean changes (95%CI) in blood pressure, glucose and BMI at follow-up by baseline pressure and intervention status *.

Outcomes	6 Months			12 Months			24 Months		
	Intervention	Control	Difference	Intervention	Control	Difference	Intervention	Control	Difference
All participants									
SBP (mmHg)	−2.9 (−4.5 ~ −1.4)	2.5 (1.1 ~ 3.9)	−5.4 (−7.5 ~ −3.4)	−4.9 (−6.8 ~ −3.0)	2.4 (0.8 ~ 4.0)	−7.3 (−9.7 ~ −4.9)	0.5 (−1.5 ~ 2.5)	4.4 (2.6 ~ 6.2)	−3.9 (−6.6 ~ −1.2)
DBP (mmHg)	−1.4 (−2.4 ~ −0.3)	2.0 (1.0 ~ 2.9)	−3.4 (−4.7 ~ −2.0)	−1.9 (−3.2 ~ −0.7)	1.9 (0.8 ~ 2.9)	−3.8 (−5.4 ~ −2.2)	0.8 (−0.4 ~ 2.1)	3.2 (2.0 ~ 4.3)	−2.3 (−4.0 ~ −0.6)
FPG (mmol/L)	−0.49 (−0.64 ~ −0.34)	−0.11 (−0.24 ~ 0.03)	−0.38 (−0.58 ~ −0.19)	−0.59 (−0.77 ~ −0.41)	0.08 (−0.06 ~ 0.23)	−0.67 (−0.90 ~ −0.45)	−0.16 (−0.34 ~ 0.02)	0.07 (−0.09 ~ 0.23)	−0.23 (−0.47 ~ 0.01)
BMI (kg/m^2^)	−0.25 (−0.36 ~ −0.14)	0.03 (−0.06 ~ 0.12)	−0.28 (−0.41 ~ −0.15)	−0.33 (−0.47 ~ −0.19)	0.17 (0.06 ~ 0.28)	−0.50 (−0.67 ~ −0.34)	−0.08 (−0.23 ~ 0.06)	0.30 (0.17 ~ 0.43)	−0.38 (−0.58 ~ −0.19)
Nonhypertensive									
SBP (mmHg)	−1.2 (−3.0 ~ 0.6)	3.0 (1.5 ~ 4.6)	−4.2 (−6.6 ~ −1.9)	−3.8 (−6.1 ~ −1.5)	1.7(−0.1 ~ 3.5)	−5.5 (−8.5 ~ −2.6)	1.5 (−1.0 ~ 3.9)	6.2 (4.0 ~ 8.3)	−4.7 (−7.9 ~ −1.5)
DBP (mmHg)	0.1 (−1.1 ~ 1.3)	2.8 (1.8 ~ 3.8)	−2.7 (−4.2 ~ −1.5)	−0.9 (−2.4 ~ 0.6)	2.7 (1.6 ~ 3.8)	−3.6 (−5.4 ~ −1.8)	2.2 (0.7 ~ 3.7)	4.4 (3.1 ~ 5.7)	−2.2 (−4.2 ~ −0.2)
FPG (mmol/L)	−0.47 (−0.66 ~ −0.27)	−0.08 (−0.25 ~ 0.09)	−0.39 (−0.64 ~ −0.13)	−0.59 (−0.83 ~ −0.35)	0.09 (−0.09 ~ 0.26)	−0.68 (−0.97 ~ −0.38)	−0.33 (−0.55 ~ −0.10)	0.07 (−0.12 ~ 0.26)	−0.40 (−0.70 ~ −0.10)
BMI (kg/m^2^)	−0.28 (−0.42 ~ −0.14)	−0.01 (−0.12 ~ 0.10)	−0.27 (−0.45 ~ −0.09)	−0.38 (−0.56 ~ −0.20)	0.16 (0.03 ~ 0.29)	−0.54 (−0.75 ~ −0.32)	−0.06 (−0.24 ~ 0.12)	0.28 (0.14 ~ 0.43)	−0.34 (−0.58 ~ −0.11)
Hypertensive									
SBP (mmHg)	−5.6 (−8.1 ~ −3.0)	1.9 (−0.6 ~ 4.6)	−7.5 (−10.9 ~ −4.2)	−7.3 (−10.3 ~ −4.3)	3.6 (0.7 ~ 6.4)	−10.9 (−14.7 ~ −7.0)	−0.9 (−4.2 ~ 2.4)	3.2 (0.1 ~ 6.3)	−4.1 (−8.5 ~ 0.4)
DBP (mmHg)	−3.5 (−5.3 ~ −1.8)	0.9 (−0.9 ~ 2.7)	−4.4 (−6.7 ~ −2.1)	−3.8 (−5.8 ~ −1.9)	0.9 (−1.0 ~ 2.8)	−4.7 (−7.3 ~ −2.2)	−1.1 (−3.2 ~ 1.0)	1.9 (0 ~ 3.9)	−3.0 (−5.9 ~ −0.2)
FPG (mmol/L)	−0.51 (−0.73 ~ −0.29)	−0.13 (−0.35 ~ 0.10)	−0.38 (−0.67 ~ −0.09)	−0.61 (−0.88 ~ −0.35)	0.08 (−0.18 ~ 0.33)	−0.69 (−1.03 ~ −0.35)	0.01 (−0.29 ~ 0.31)	0.05 (−0.23 ~ 0.33)	−0.04 (−0.44 ~ 0.37)
BMI (kg/m^2^)	−0.23 (−0.39 ~ −0.07)	0.05 (−0.11 ~ 0.21)	−0.28 (−0.48 ~ −0.08)	−0.31 (−0.52 ~ −0.10)	0.18 (−0.01 ~ 0.37)	−0.49 (−0.75 ~ −0.22)	−0.14 (−0.38 ~ 0.10)	0.29 (0.07 ~ 0.52)	−0.43 (−0.76 ~ −0.11)

* Estimates are based on a linear mixed-effects regression adjusted for sex, age, education, and baseline values.

**Table 5:** Odds ratios and mean changes (95%CI) in health-related behaviour at follow-up in the intervention and control groups.

Outcomes	6 months			12 months			24 months		
	Intervention	Control	Odds Ratio or Difference	Intervention	Control	Odds Ratio or Difference	Intervention	Control	Odds Ratio or Difference
Current smoker *	0.37 (0.17 ~ 0.79)	1.07 (0.43 ~ 2.66)	0.34 (0.10 ~ 1.14)	0.45 (0.19 ~ 1.10)	0.75 (0.28 ~ 2.02)	0.60 (0.16 ~ 2.27)	0.62 (0.26 ~ 1.48)	0.67 (0.24 ~ 1.88)	0.93 (0.24 ~ 3.60)
Alcohol use *	0.23 (0.10 ~ 0.51)	0.73 (0.36 ~ 1.49)	0.31 (0.11 ~ 0.90)	0.38 (0.17 ~ 0.85)	0.92 (0.44 ~ 1.89)	0.41 (0.14 ~ 1.23)	0.35 (0.15 ~ 0.83)	0.63 (0.29 ~ 1.39)	0.56 (0.17 ~ 1.80)
Salt (g/d) ^†^	−2.39 (−2.67 ~ −2.11)	−1.07 (−1.35 ~ −0.79)	−1.32 (−1.72 ~ −0.92)	−3.11 (−3.44 ~ −2.79)	−1.02 (−1.35 ~ −0.70)	−2.09 (−2.55 ~ −1.63)	−2.51 (−2.89 ~ −2.14)	0.07 (−0.30 ~ 0.44)	−2.58 (−3.11 ~ −2.06)
Cooking oil(g/d) ^†^	−4.07 (−4.94 ~ −3.20)	1.09 (0.21 ~ 1.97)	−5.16 (−6.41 ~ −3.93)	−5.38 (−6.36 ~ −4.39)	0.22 (−0.75 ~ 1.20)	−5.60 (−6.99 ~ −4.21)	−3.79 (−4.88 ~ −2.70)	2.83 (1.77 ~ 3.90)	−6.62 (−8.14 ~ −5.10)
Vegetables (g/d) ^†^	65.6 (55.6 ~ 75.5)	12.2 (2.1 ~ 22.3)	53.4 (39.2 ~ 67.6)	109.2 (97.6 ~ 120.7)	8.4 (−3.0 ~ 19.7)	100.8 (84.6 ~ 117.0)	82.3 (69.4 ~ 95.2)	8.1 (−4.4 ~ 20.7)	74.2 (56.1 ~ 92.2)
Fruits (g/d) ^†^	12.0 (5.1 ~ 18.9)	9.1 (2.1 ~ 16.0)	2.9 (−6.8 ~ 12.7)	15.0 (7.1 ~ 22.9)	5.8 (−2.0 ~ 13.6)	9.2 (−1.9 ~ 20.3)	10.4 (1.6 ~ 19.1)	4.2 (−4.4 ~ 12.7)	6.2 (−6.0 ~ 18.4)

* Estimates are odds ratios from a logistic mixed-effects regression adjusted for sex, age, education and baseline values. Values less than 1 reflect improvement compared with baseline values or the control group, and values higher than 1 reflect deterioration; ^†^ Estimates are based on a linear mixed-effects regression adjusted for sex, age, education and baseline values.

### 3.6. Lifestyle Changes

Table 5 depicts the changes in lifestyle made by the intervention group. Compared with the control group participants, those in the intervention group demonstrated sustainable decreases in self-reported intake of salt (6 months: −1.32 g/d; 12 months: −2.09 g/d; 24 months: −2.58 g/d; all *p* < 0.001) and cooking oil (6 months: −5.16 g/d; 12 months: −5.60 g/d; 24 months: −6.62 g/d; all *p* < 0.001) over the follow-up period. The intervention group also showed a significant increase in vegetable intake from baseline at all three follow-up visits (6 months: 53.4 g/d; 12 months: 100.8 g/d; 24 months: 74.2 g/d; all *p* < 0.01). Although significant improvements were observed in alcohol use and fruit intake in the intervention group at follow-up, only the change in alcohol use differed significantly from the control group at 6 months. Changes in current smoking did not differ between groups. Participants in the intervention group increased their activity levels one to two-fold during the follow-up period, and as against there was a slight increase in the control group. Medication use among hypertensive patients improved in both groups at the follow-up visits, but those in the intervention group were more likely to adhere to their pharmacological treatment at each follow-up (Table 3).

## 4. Discussion

These results suggest that comprehensive lifestyle interventions can be implemented effectively using a community-based approach, and thus achieved long-term improvements in BP and glucose in the middle-aged and older population. Individuals who received the lifestyle intervention made and sustained behavioural changes and weight loss over the subsequent 12 months. Our findings are of important public health relevance because reductions in BP and glucose and the adoptions of a long-term healthy lifestyle are associated with reduced CVD and mortality risks [24,25,26].

The intervention was delivered in both group and individual formats by field health workers in a community setting and was supervised by the research team. Consequently, the implementation of the programme minimised the research resources and enhanced the responsibilities of the community workers. The results demonstrate that delivery of lifestyle interventions by community health staff is a promising vehicle for the primary prevention of CVD. Despite the considerable effectiveness, the delivery of such an intensive lifestyle intervention require involvement by community organizations with great expertise and resources for offering intensive programmes, which could be highly difficult for health workers in an under-resourced community setting. In light of recent national data showing an alarming epidemic of HT and DM, delivery of lifestyle interventions through the community-based healthcare infrastructure warrants further study as a model for the implementation of effective strategies for combating CVD and other chronic diseases in under-resourced settings.

Several lifestyle changes, included dietary intakes, physical exercise and medication adherence, responded favorably to the intervention. The observed effect may be attributable to the theory-driven design (HBM) and the use of modern behavioural approaches (e.g., self-monitoring, feedback and reinforcement). Previous evidence has shown that theory-based interventions are more likely to be effective than atheoretical approaches in controlling the behavioral risk factors of CVD [27,28]. Our finding supports current recommendations on that applying behavioral theories is an important part of the design of interventions for lifestyle improvement.

Previous reports have documented that maintaining weight loss over time is a challenge [29]. In this study, the improvements in BMI were not fully sustained at the 12- and 24-month assessments. The findings were consistent with other studies [30,31], and reflect the difficulties of sustaining weight reduction. Therefore, post-intervention services for maintaining weight loss, or at least preventing additional weight gain, should be developed.

The 24-month duration of the study was designed to evaluate the effectiveness and sustainability of the intervention. In the intervention group, the greatest reductions in BP and glucose were observed at 12 months; beneficial trends in these outcomes were maintained, but attenuated, at 24 months. Similar results for BP-related outcomes have been reported in several clinical trials [30,31,32]. The attenuation of improvements at the final visit may have been due to a decline in participants’ adherence to lifestyle changes over the longer term, with a consequent diminution of the intervention’s effects on weight loss, daily intake of vegetables, physical exercise and medication adherence.

Another interesting finding was the greater BP-lowering effect observed in hypertensive compared with non-hypertensive individuals. One probable explanation is that participants with HT received more intervention sessions. It is also possible that hypertensive patients were better at adhering to their lifestyle goals due to their poor self-perceived health status. Thus, further studies are needed to understand how perceptions of disease risk influence individuals’ receptivity to lifestyle interventions.

Despite the significant results, the generalizability of the findings should be interpreted cautiously because it involved only two typical communities from a single district, and participants were randomly assigned to either a community-based lifestyle intervention or conventional health education. This study design was chosen to minimise intervention contamination and potential bias due to allocation awareness. However, the group randomization may have increased the possibility of unmeasured confounding variables. Furthermore, the lack of blinding could result in bias such as overestimation of the impact of the intervention and better documentation of lifestyle intervention delivered than in conventional care. In addition, since our recruitment was conducted after the randomisation, it might engage lead to refusal of participation if the potential participants were assigned to the “un-preferred” group and therefore resulted in selection bias. Considering that this was a pilot feasibility study, a larger study with greater power is needed to evaluate the intervention in China.

There were several limitations to the study. First, the 24-month follow-up had insufficient power to determine the long-term sustainability of BP and glucose changes and the effect of the interventions on cardiovascular outcomes. However, even a small decline in the mean BP of a population is associated with fewer deaths [33,34]; Second, 24.5% and 31% of participants failed to attend the follow-up visit at 12 and 24 months, although this was balanced between two groups. The analyses were based on the intention-to-treat principle and the completion population, respectively. Both analyses yielded consistent results, indicating that our findings were robust; Finally, the design precluded us from examining which component within the multi-component intervention drove the beneficial effects because the control group did not receive any intervention. However, this was not an aim of this trial, which was designed to assess a comprehensive health promotion approach consisting of various components to encourage the adoption of healthy lifestyles. Further analyses linking process and outcome evaluations may be able to determine the differential effects of specific intervention components.

## 5. Conclusions

This study shows that community-based lifestyle interventions delivered by trained field health workers may be a potential solution for combating HT and DM among middle-aged and older adults in an under-resourced urban setting. Further larger powered studies are needed to evaluate the effectiveness of community-based models for lifestyle promotion on cardiometabolic risk prevention in China.

## Appendix

**Table A1:** Sensitivity analyses based on the completion population *****.

Outcomes	6 Months			12 Months			24 Months		
	Intervention	Control	Difference	Intervention	Control	Difference	Intervention	Control	Difference
**All participants**									
SBP (mmHg)	−2.2 (−4.4 ~ −0.1)	4.0 (2.1 ~ 5.8)	−6.2 (−8.9 ~ −3.5)	−3.7 (−6.2 ~ −1.3)	3.0 (1.0 ~ 4.9)	−6.7 (−9.7 ~ −3.7)	1.2 (−1.2 ~ 3.7)	5.3 (3.2 ~ 7.5)	−4.1 (−7.4 ~ −0.9)
DBP (mmHg)	−1.6 (−3.1 ~ −0.2)	2.6 (1.3 ~ 3.9)	−4.2 (−6.1 ~ −2.4)	−1.9 (−3.5 ~ −0.2)	1.9 (0.6 ~ 3.2)	−3.8 (−5.8 ~ −1.8)	0.5 (−1.2 ~ 2.1)	3.3 (1.9 ~ 4.7)	−2.8 (−5.0 ~ −0.7)
FPG (mmol/L)	−0.51 (−0.72 ~ −0.31)	−0.11 (−0.29 ~ 0.06)	−0.40 (−0.66 ~ −0.15)	−0.60 (−0.82 ~ −0.38)	0.07 (−0.10 ~ 0.25)	−0.67 (−0.94 ~ −0.41)	−0.18 (−0.39 ~ 0.03)	0.09 (−0.08 ~ 0.27)	−0.27 (−0.54 ~ 0)
BMI (kg/m^2^)	−0.30 (−0.44 ~ −0.15)	0.09 (−0.03 ~ 0.21)	−0.39 (−0.56 ~ −0.21)	−0.40 (−0.57 ~ −0.22)	0.18 (0.04 ~ 0.32)	−0.58 (−0.79 ~ −0.36)	−0.17 (−0.36 ~ 0.01)	0.32 (0.16 ~ 0.48)	−0.49 (−0.74 ~ −0.25)
**Nonhypertensive**									
SBP (mmHg)	−2.2 (−4.7 ~ 0.2)	4.2 (2.2 ~ 6.3)	−6.4 (−9.7 ~ −3.2)	−4.6 (−7.5 ~ −1.7)	2.2 (−0.1 ~ 4.4)	−6.8 (−10.4 ~ −3.2)	0.6 (−2.3 ~ 3.5)	7.0 (4.5 ~ 9.5)	−6.4 (−10.3 ~ −2.5)
DBP (mmHg)	−0.4 (−1.9 ~ 1.2)	3.3 (2.0 ~ 4.7)	−3.7 (−5.7 ~ −1.6)	−0.9 (−2.7 ~ 0.9)	2.8 (1.4 ~ 4.2)	−3.7 (−6.0 ~ −1.4)	1.8 (−0.1 ~ 3.6)	4.6 (3.0 ~ 6.1)	−2.8 (−5.2 ~ −0.3)
FPG (mmol/L)	−0.47 (−0.75 ~ −0.18)	0 (−0.23 ~ 0.23)	−0.47 (−0.83 ~ −0.10)	−0.54 (−0.85 ~ −0.22)	0.09 (−0.14 ~ 0.32)	−0.63 (−1.01 ~ −0.24)	−0.31 (−0.59 ~ −0.02)	0.07 (−0.16 ~ 0.31)	−0.38 (−0.76 ~ 0)
BMI (kg/m^2^)	−0.37 (−0.55 ~ −0.19)	0.06 (−0.09 ~ 0.20)	−0.43 (−0.66 ~ −0.20)	−0.47 (−0.69 ~ −0.25)	0.15 (−0.01 ~ 0.30)	−0.62 (−0.88 ~ −0.36)	−0.18 (−0.40 ~ 0.03)	0.29 (0.12 ~ 0.46)	−0.47 (−0.75 ~ −0.20)
**Hypertensive**									
SBP (mmHg)	−3.6 (−7.1 ~ 0)	3.9 (0.7 ~ 7.2)	−7.5 (−11.9 ~ −3.1)	−4.7 (−8.6 ~ −0.7)	4.2 (0.8 ~ 7.6)	−8.9 (−13.7 ~ −4.0)	−0.9 (−3.3 ~ 5.0)	4.3 (0.7 ~ 8.0)	−3.4 (−8.9 ~ 2.0)
DBP (mmHg)	−3.9 (−6.5 ~ −1.4)	1.7 (−0.7 ~ 4.0)	−5.6 (−8.8 ~ −2.4)	−4.2 (−6.9 ~ −1.4)	0.8 (−1.5 ~ 3.2)	−5.0 (−8.3 ~ −1.6)	−2.0 (−4.7 ~ 0.8)	2.1 (−0.3 ~ 4.5)	−4.1 (−7.7 ~ −0.5)
FPG (mmol/L)	−0.57 (−0.85 ~ −0.29)	−0.19 (−0.44 ~ 0.06)	−0.38 (−0.72 ~ −0.04)	−0.70 (−1.02 ~ −0.39)	0.07 (−0.19 ~ 0.34)	−0.77 (−1.16 ~ −0.39)	−0.08 (−0.42 ~ 0.25)	0.14 (−0.16 ~ 0.43)	−0.22 (−0.66 ~ 0.21)
BMI (kg/m^2^)	−0.23 (−0.46 ~ 0)	0.10 (−0.10 ~ 0.30)	−0.33 (−0.60 ~ −0.06)	−0.32 (−0.60 ~ −0.03)	0.20 (−0.03 ~ 0.44)	−0.52 (−0.86 ~ −0.17)	−0.16 (−0.49 ~ 0.16)	0.35 (0.06 ~ 0.63)	−0.51 (−0.94 ~ −0.09)

***** Estimates are based on a linear mixed-effects regression adjusted for sex, age, education, and baseline values.
